# Effect of modulating braille dot height on reading regressions

**DOI:** 10.1371/journal.pone.0214799

**Published:** 2019-04-17

**Authors:** Daisy Lei, Natalie N. Stepien-Bernabe, Valerie S. Morash, Manfred MacKeben

**Affiliations:** 1 The Smith-Kettlewell Eye Research Institute, San Francisco, California, United States of America; 2 Vision Science Program, School of Optometry, University of California, Berkeley, California, United States of America; Nagoya University, JAPAN

## Abstract

It is well known that people who read print or braille sometimes make eye or finger movements against the reading direction. The way these regressions are elicited has been studied in detail by manipulating linguistic aspects of the reading material. Actually, it has been shown that reducing the physical intensity or clarity of the visual input signal can also lead to increased regressions during reading. We asked whether the same might be true in the haptic realm while reading braille. We set the height of braille dots at three different levels (high, medium, and low) and asked adult blind, practiced braille readers to read standardized texts without any repetition of content. The results show that setting the braille dot height near the tactile threshold significantly increased the frequency of regressive finger movements. Additionally, at the lowest braille dot height, braille reading speed significantly diminished. These effects did not occur at braille dot heights that were closer to the height of standard braille (medium and high). We tentatively conclude that this effect may be due to a heightened sense of uncertainty elicited by perception near the threshold that seems to be common to the reading process, independent of the sensory input modality. Furthermore, the described effect may be a feature of a brain area that contributes to the reading process mediated by vision as well as touch.

## Introduction

Reading constitutes the sequential acquisition of information that can be imparted through a variety of media. In visual reading, the eyes wander across visible text by a series of fast (saccadic) eye movements mainly along the lines of text [1–4]. The saccades are interspersed by fixations, with mean duration of 275–325 ms for oral reading and 225–350 ms for silent reading, during which the actual process of acquiring information occurs [5–7].

The rich body of knowledge of the visual reading process has led to the formulation of several theoretical models of reading and eye movements. They all addressed the task of recognizing letters in the presence of noise, identifying words and making the appropriate eye movements to the next word.

Most prominent was the creation of “Mr. Chips”, based on an ideal observer strategy [8–9], while others have used Bayesian observer models that derive the inference from letter data: “E-Z Reader” [10,11], “SWIFT” [12,13], a “rational model” [14] (which particularly focused on the occurrence of between-word regressions). Others have also hypothesized that regressions are mainly determined by low-level visuomotor processes (e.g., word skipping due to oculomotor aiming errors) [15]. An additional influence of different reading strategies cannot be ruled out, but it is beyond the purview of this study. For a detailed discussion of different reading and eye movement models see [16].

Regarding eye movements, the models consider different functional states to choose between three actions: (a) continuing to fixate a currently-fixated position, (b) initiating a saccade to a new position, or (c) stopping to read a sentence. One parameter to differentiate between these options is word and sentence comprehension. Option (b) can also include the decision whether to move the eye forward or backward to a previously read word [14].

In the tactile reading of braille the process is similar, only that the fingers wander continuously over a series of raised dots that are grouped to denote letters or small groups of letters; but, in this case, acquiring information happens during movement [17].

There is an objective aspect of the reading process that depends on the quality of the physical input signal. The aim of this study was to investigate the role of physical input quality on braille reading. Our approach addresses the fact that the signal can be degraded by physical properties, in the visual as well as the tactile realms. Visual input can be degraded by very low contrast; tactile input can be degraded by reduced braille dot heights.

There is also a subjective and individual aspect that determines the quality of the sensory input by a change of sensitivity. Visual sensitivity can be diminished by an eye disease, tactile sensitivity by age. Both these components affect the ability of the individual sensory organ to derive an unequivocal message from the signal received from the input medium. We focus here on the objective aspect of reading, because it is fundamental for solving the task and has not received enough attention.

In reading, most eye and finger movements are progressive; that is, they travel in the reading direction and, thus with the flow of grammatical and semantic content. It has long been known, however, that not all movements are progressive [18–20]. According to Rayner [6], 10–15% of saccades by readers of English print are regressions that lead either just a few letters back, or to a previously read word.

Additionally, it has long been known that there are several features that can provoke regressions, such as lack of reading experience (especially in children) and unfamiliarity with the vocabulary or text layout [18, 21]. Visuomotor factors have also been shown to influence regression frequency [22–24]. In general, Bayle [25] conducted an early eye movement analysis and recognized the importance of the quality of the input signal and postulated that regressions appear “when perception is inadequate”. Later research has confirmed these early notions [26,27] and added many linguistic aspects of the text as possible causes [28, 23, 29–30]. Indeed, linguistic manipulations of the text can have the same effect on reading braille and increase the likelihood of regressive hand movements [31, 17, 32–35]. The kinematic properties of reading hand movements and regressive hand movements are not qualitatively different [36].

Experiments on reading print under extreme conditions, like very low contrast, have shown that, as the quality of the physical input diminished near the sensory threshold, there is an increased uncertainty about the input signal. For instance, it has been found that reducing brightness contrast of printed text to near the contrast sensitivity threshold leads to more frequent regressive saccades [37]. The same effect occurs if the sensory apparatus is weakened by a pathological condition, as is the case with an eye disease [38] or with abnormal signal processing in the brain due to amblyopia [39].

Considering that the effects of linguistic manipulations occur independent of medium, one might assume that the common reason underlying regressions could be that the reader feels unsure about the meaning of the recently read text [40]. We ask, therefore, whether the role of input quality is a domain-specific characteristic of the visual system, or if this influence also exists during haptic reading. Particularly, we investigated whether diminishing the intensity of tactile input can increase the likelihood of regressive hand movements while reading braille. Consequently, the aim of this project was to perform experiments with tactile input of different physical levels of quality on adult, experienced braille readers.

There are several methods of measuring braille reading via tracking finger movements. For example, researchers have recorded finger movements using a video recorder and then coding the observed finger movements offline. More recent methods include using an infrared camera to track finger movements (for a review, see [41]). In this study, however, we used a relatively novel alternative method developed in our lab to record finger movements while participants read braille. Details on this finger tracking method are provided in the Procedures sections below.

Based on pilot data and evidence from previous vision research, we hypothesized that braille reading regressions could occur due to simply diminishing the physical quality of the tactile input without manipulating any linguistic variables. If true, this finding would imply that reading regressions can result from uncertainty about recently-read text without linguistic manipulations and independent of the sensory modality. This concept is explained in detail in the Discussion.

## Methods

### Participants

We report data from 12 blind adults (age *M* = 41 years, *SD* = 13.94, range = 22 to 70 years, eight female). Only fluent braille readers were included in this study. An additional six adults were excluded from further analysis: two because they read braille in the typical braille dot height too slowly (their reading times were more than 1.5 times the interquartile range above the third quartile (Q3), which are outliers according to the IQR outlier rule), one because of excessive reading mistakes, one because of a hand injury that impacted braille reading, and two because of experiment error. All participants had begun receiving braille instruction between the ages of 4–8 years. Participants responded to the Edinburgh Handedness Inventory [42], a questionnaire that assesses hand preference for everyday items or tasks. Their handedness scores and their preferred hand during one-handed braille reading were unrelated (Kendall’s Tau-b = 0.04, *p* = .936).

There are many ways one can read braille text; individuals may use one hand or two hands. Additionally, in individuals who habitually read braille with two hands, there are a variety of movement patterns they may use, e.g., reading with both index fingers side by side across the entire page, or reading part of the line with the left hand, and part of the line of text with the right hand [17]. All but one of our participants preferred to read braille using two hands. For these experiments, however, we asked participants to use only one hand, although this may have reduced the reading speed of habitual two-handed braille readers. In the discussion of the experimental results, it will be inevitable to make comparisons between reading braille and reading print. Any such comparison would be unfair if we juxtaposed reading print by two-handed braille reading, since the latter allows independent hand movements, while reading print does not include independent movements of the eyes. Hence, this report only discusses braille reading movements observed during one-handed reading of braille.

All participants came from the San Francisco Bay Area and were recruited through an email list and word-of-mouth. They received monetary compensation for their time. The Smith-Kettlewell Eye Research Institute Institutional Review Board approved the experimental procedures, and all participants provided informed written consent prior to their enrollment.

### Reading material

To control for linguistic complexity as a potential factor for causing regressions, we ensured that texts were of the same reading level and of similar length and syntactic complexity by using the International Reading Speed Texts (IReSTs), a set of standardized texts designed for assessing reading performance in teenagers and adults [43]. These texts allowed us to control for linguistic complexity as a potential factor for causing regressions, since they were intentionally designed to have the same reading ability level (sixth grade), similar length (830 ± 2 characters), high frequency words, and varied but comparable linguistic complexity [43, 44].

Six English IReST paragraphs were used as experimental stimuli (titled “mice”, “beaver”, “prey”, “trees”, “desert”, and “island”), and one was selected for a practice trial (titled “colors”). The texts received minor edits to conform to American English spelling and word usage, and they were translated into American English contracted braille. Contracted braille is not a direct letter-to-symbol mapping with printed text. The most important difference is that braille symbols and symbol groups are used to replace a mix of single letters and punctuation, parts of words (e.g., “ea” and “ount”), and whole words (e.g., “enough” and “were”). Spaces between some words are removed in contracted braille. Due to the larger footprint of braille, an IReST paragraph occupied a full 11.5 x 11 inch (29.21 x 27.94 cm) standard braille page. Each sheet was used only once, so that repeated use would not lower the height of the dots.

### Stimuli

Each text was printed on a standard braille sheet using a VP Max embosser (ViewPlus Technologies, Inc.). While the Library of Congress [45] recommends that braille dots on embossed paper be 0.48 mm tall, the maximum and default braille dot height printed by the VP Max embosser was 0.38 mm, which was used as the “high” condition. Because the high braille dot height is the default height of the braille embosser we used, this can also be considered the “standard” height for braille embossed on paper. Three braille dot heights were printed by the VP Max embosser by manipulating the “opacity” setting in Adobe Photoshop (Adobe Systems Inc., San Jose, CA). Braille dots were printed in one of three heights: high *M* = 0.38 mm (*SE* = 0.01 mm), medium *M* = 0.18 mm (*SE* = 0.01 mm), or low *M* = 0.04 mm (*SE* = 0.01 mm). These values were determined by 18 measurements of each height, taken from used and unused stimuli on different positions on the braille page (neither variable statistically affected heights), using a 10x magnifying reticle.

### Procedures

**Fig 1:**
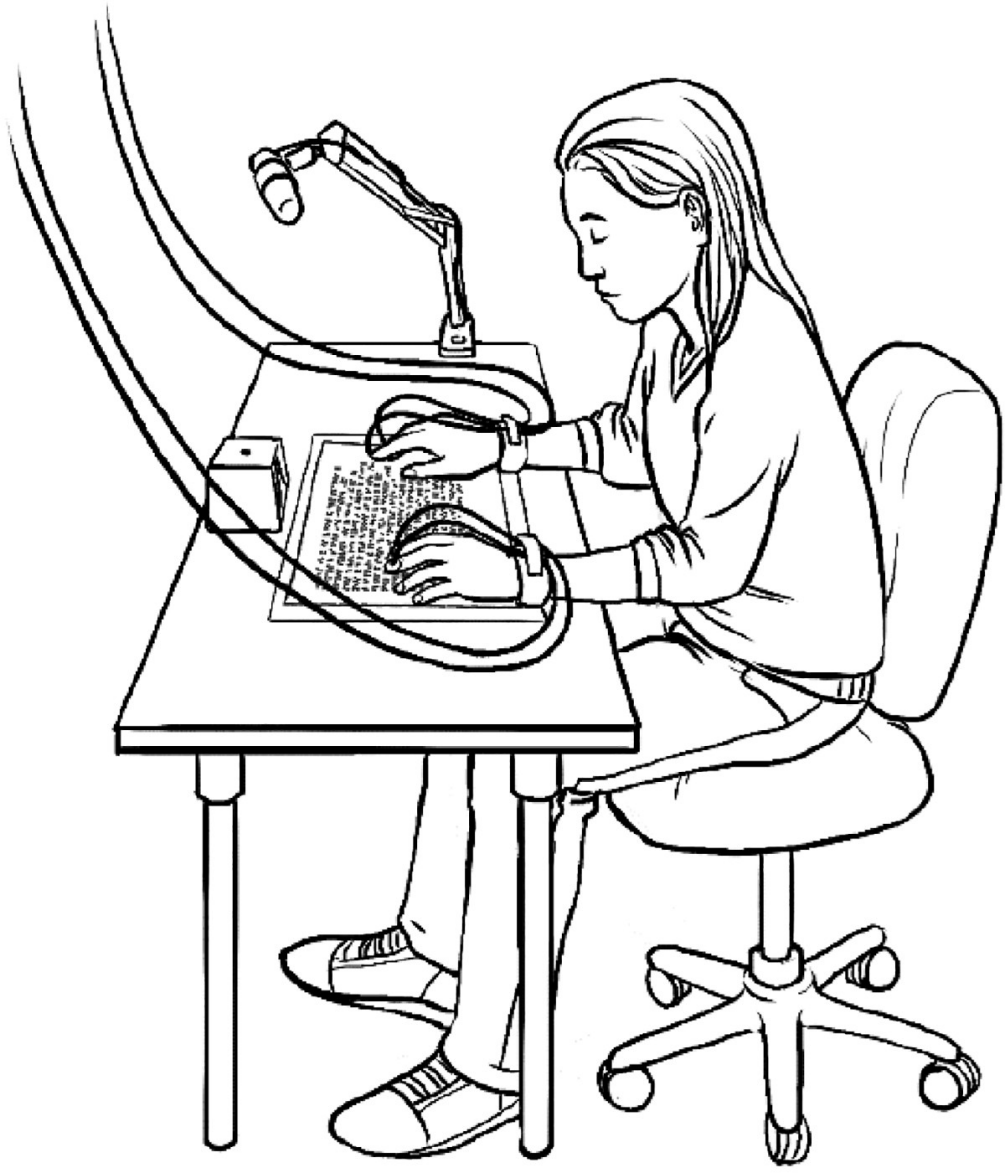
Experimental setup.

For the duration of the experimental procedures, participants were seated at a table on which braille sheets were presented in a fixed location (Fig 1). A microphone above the table captured their verbal responses. Participants had electromagnetic sensors taped to the tops of their index and middle fingernails, which tracked finger positions at 240 Hz (3DG trakSTAR system by Northern Digital Inc.; 2.0 mm sensors with 1.4 mm root mean square position error). They were asked to read the braille texts aloud and were informed that the texts would vary in braille dot height. The decision to have the participants read the texts aloud was made to help with detecting and recognizing reading errors. Indeed, reading visual print aloud invokes more frequent regressions than silent reading [46], which may also be true for reading braille.

Participants completed one practice trial with high braille dots to familiarize them with the procedures. They were allowed to read using the hand and fingers they preferred. Following the practice trial, they read three texts with one hand. Assignment of text to braille dot height condition was pseudorandom, with the constraint that each text appeared an equal number of times in each condition across the 12 participants. Specifically, each participant read three different texts, each at a different dot height (low, medium, and high). Across the 12 participants, each text was read once at each dot height condition. The reading time for a page of text ranged from 66 to 206 seconds (equivalent to 136 and 44 words per minute).

### Data processing

Index finger movements of the reading hand were labeled either as forward movements, backward movements, or line returns. Forward movements are left to right movements, indicating reading movements. Backward movements are right to left movements to a previously-read word, or to a word on the previous line. Unlike visual reading of text characterized by a series of fixations and saccades, braille reading is characterized by continuous hand movements and is typically exhaustive reading of all characters on the page [17]. Hence, backward movements of at least a quarter of an inch, or the width of a braille cell, were labeled as regressions. A regression from line *n* to a previous line (*n-1*) was labeled as a regression from line *n*.

## Analyses

All analyses were conducted in R (The R Foundation for Statistical Computing, Vienna, Austria, version 3.3.3). Specifically, the lme4 package [47] was used for the general and generalized mixed effects modeling, and the emmeans package [48] was used for the post-hoc pairwise comparisons of the estimated marginal means. All post-hoc tests were corrected for multiple comparisons using the Holm-Bonferroni procedure for the 95% confidence level adjustment and *p*-value adjustment [49].

### Reading rates

Average reading rates in words per minute were calculated as the number of words in each text divided by the participants’ reading times in minutes. Because of the wide age span of our cohort (22 to 70 years), we had to consider the possibility that there may be differences in reading speed based on the documented decline of tactile sensitivity with age [50]. In our sample, however, age and reading speed were not significantly correlated (Pearson’s correlation *r* = .27, *p* = .398).

Otherwise, reading rates were analyzed using a general mixed effects model with random intercept effect of participant and a fixed effect of braille dot height. Likelihood ratio tests were used to compare the different models’ goodness of fit, followed by post-hoc pairwise comparisons of the estimated marginal means on braille dot height.

### Regressive movements

Unlike with reading print, where information is acquired during fixations, braille reading requires individuals to move their fingers across the raised dots to acquire information. It is possible that there will be multiple left to right movements to reread the same word. To better interpret regressions in braille reading, we differentiated between *primary regressions*, the first regressive movement to a particular word or a series of words, and *repeat regressions*, all other regressive movements to the same word or a series of words. To include the repeat regressions with the primary regressions would superficially inflate the unique number of regressions.

Accordingly, regressions were investigated using four separate metrics. The first two metrics were the number of primary regressions and the number of repeat regressions. The third was the number of times each word was read using a distinct forward (left to right) movement with a length of at least 0.25 in (6.35 mm). Forward movements were separated from one another by regressive movements of at least 0.25 in (6.35 mm). If no reading regressions were made, the number of times each word was read would be one. The fourth metric was the percentage of time spent in regressive movements during each trial. The analyses for the number of primary regressions that occurred, the number of repeat regressions that occurred, and the number of times each word was read were conducted as a general (linear) mixed effects model with a random intercept effect of participant and a fixed effect of braille dot height. Likelihood ratio tests were used to compare the different models’ goodness of fit, followed by post-hoc pairwise comparisons of the estimated marginal means on braille dot height. Analyses of the percentage of time in regressive movements were conducted as generalized mixed effects models using a Gamma distribution with a random intercept effect of participant, due to violations of normality, agreement with Gamma model assumptions, and better fit based on the Akaike Information Criterion (AIC).

### Movement speeds

Finger movement speeds were compared for forward and regressive movements using a generalized mixed effects model using a Gamma distribution (due to violations of normality) with a random intercept effect of participant and fixed effects of movement direction (forward and regressive) and braille dot height. Likelihood ratio tests were used to examine which fixed effects should be included in the model and whether there was an interaction between the fixed effects, followed by a post-hoc pairwise comparison of the estimated marginal means on movement direction and braille dot height.

## Results

### Reading rates

**Fig 2:**
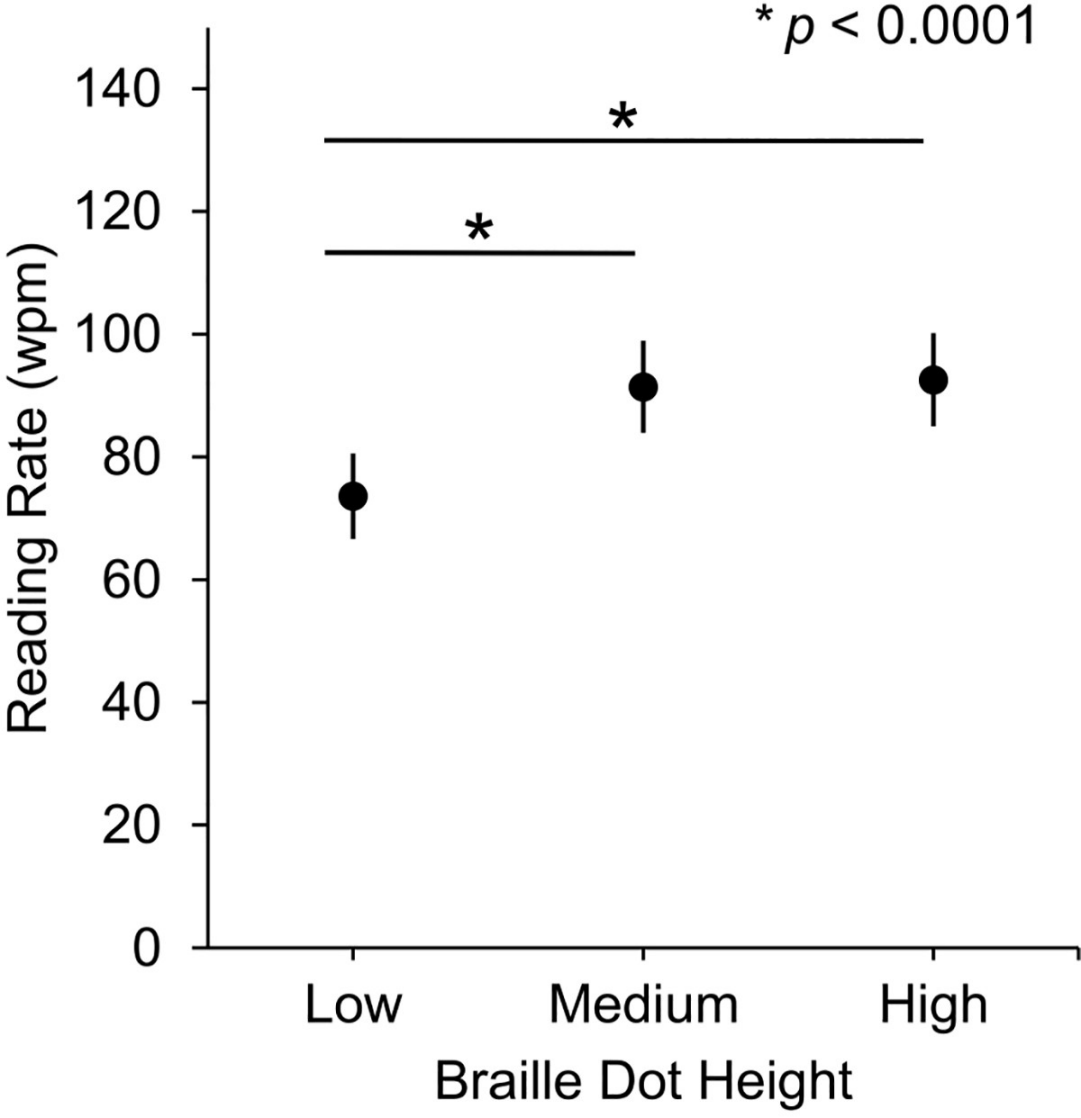
Average readings rates (with SEMs) at three braille dot heights.

Reading text embossed in medium and high braille dots was faster than reading low height braille dots. The participants’ average reading rates (Fig 2) were normally distributed and were significantly affected by braille dot height, χ^2^(2) = 85.86, *p* < .001, (see Table 1 for a statistics table of the fixed effects of the model). Post-hoc pairwise estimated marginal means analysis revealed significant differences in reading rates for low and medium braille dot heights (*p* < .0001, 95% CI [-23.97, -11.69]) and low and high braille dot heights (*p* < .0001, 95% CI [-25.12, -12.85]), but no significant difference between medium and high braille dot heights (*p* = .635, 95% CI [-7.29, 4.99]).

**Table 1:** Statistics table of the fixed effects for each model.

	*beta*	*SE*	*df*	*t*	*p*-value
**Reading Rates**					
(Intercept)	73.64	7.08	12.88	10.41	< .001
height2	17.83	2.30	24	7.76	< .001
height3	18.98	2.30	24	8.26	< .001
**Primary Regressions**					
(Intercept)	30.67	3.19	15.29	9.61	< .001
height2	-9.50	2.21	22	4.31	< .001
height3	-12.50	2.21	22	5.67	< .001
**Repeat Regressions**					
(Intercept)	14.17	2.30	19.74	6.16	< .001
height2	-6.67	2.11	22	3.16	.005
height3	-8.08	2.11	22	3.83	< .001
**Percent of Time Rereading**					
(Intercept)	-2.17	0.12	-	17.80	< .001
height2	-0.28	0.12	-	2.33	.020
height3	-0.21	0.12	-	1.78	.075

The low braille dot height is not listed in the table because it is used as a reference variable (Intercept) in the models. height2 = medium braille dot height; height3 = high braille dot height.

### Regressive movements

We found that 24.54% of all regressive movements occurred in high braille dot height, 29.04% in medium height, and 46.42% in low height. Across all regressive movements, 57.20% were within a word, 35.88% were within a line beyond a word, and 1.53% were to a previous line. On average, 1–4% of regressions were to a previously-read line (low: *M* = 2.09%, SEM = 0.98%; medium: *M* = 1.66%, SEM = 0.62%; high: *M* = 3.29%, SEM = 1.15%). The fixed effects statistics table for each model related to regressive movements are reported in Table 1.

#### Primary and repeat regressions

Some regressions (5.63%) began at an empty space in the text (rather than a braille cell), so that it was difficult to determine the start and end word of these regressions. Thus, the number of primary regressions was determined after excluding those regressions that began at an empty space. Of the remaining regressions, 71.61% were primary regressions and 28.49% were repeat regressions. Primary regressions and repeat regressions had a similar proportion of within-word regressions (61.55% and 58.25%, respectively). The average number of primary regressions were *M* = 30.67, SEM = 3.69; *M* = 21.17, SEM = 2.81; and *M* = 18.17, SEM = 3 for low, medium, and high braille dot heights respectively. The average number of repeat regressions for each braille dot height condition were low: *M* = 14.17, SEM = 3.2; medium: *M* = 7.5, SEM = 1.94; and high: *M* = 6.08, SEM = 1.37.

**Fig 3:**
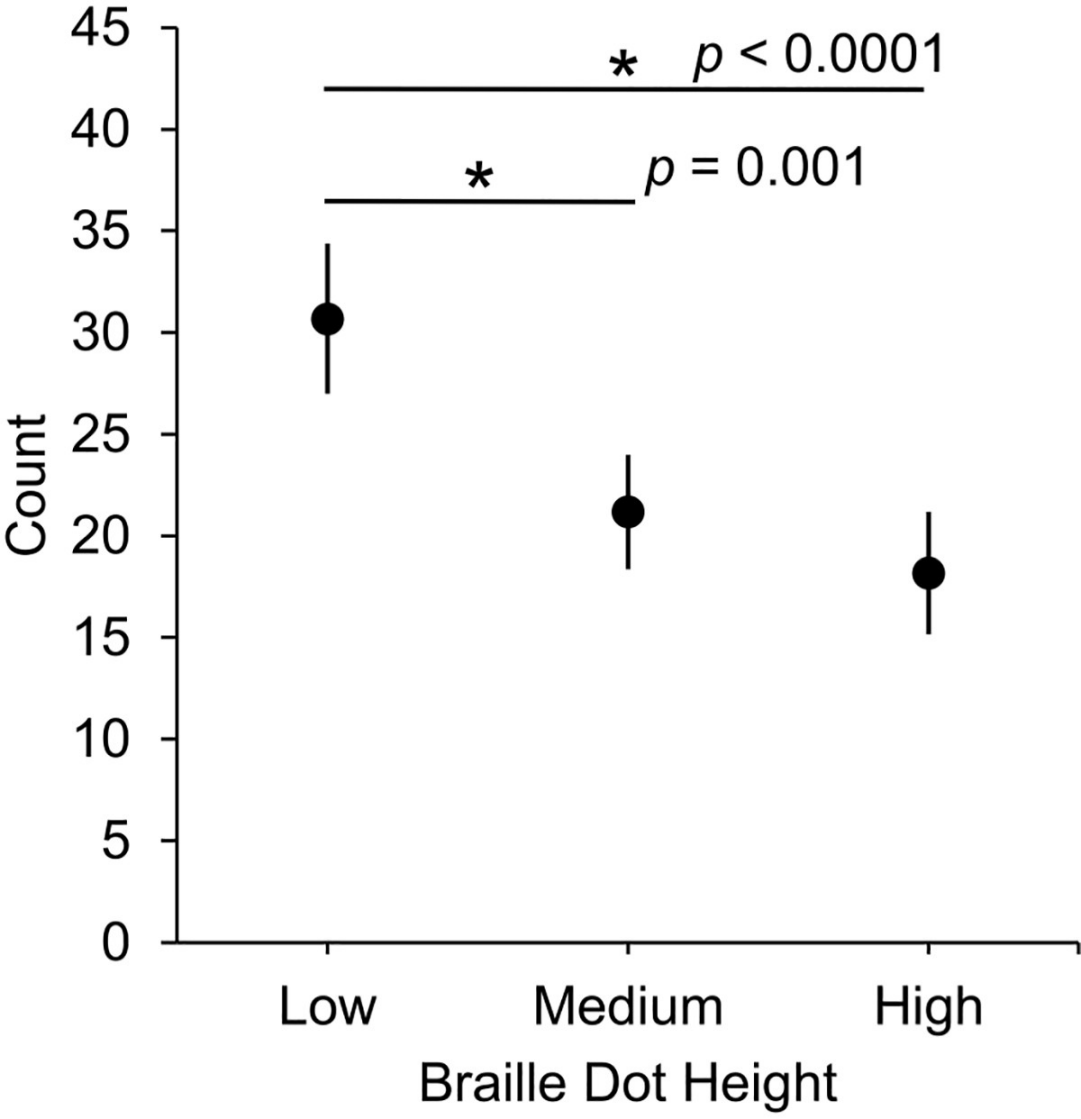
Average number of primary regressions (with SEMs) at three braille dot heights.

The number of primary regressions (Fig 3) was significantly affected by braille dot height, χ^2^(2) = 35.02, *p* < .0001. Post-hoc pairwise estimated marginal means comparisons indicated that the number of primary regressions was significantly different between low and medium braille dot heights (*p* = .001, 95% CI [3.78, 15.21]) and between low and high braille dot heights (*p* < .0001, 95% CI [6.79, 18.21]), but not significantly different between medium and high braille dot heights (*p* = .188, 95% CI [-2.71, 8.71]).

**Fig 4:**
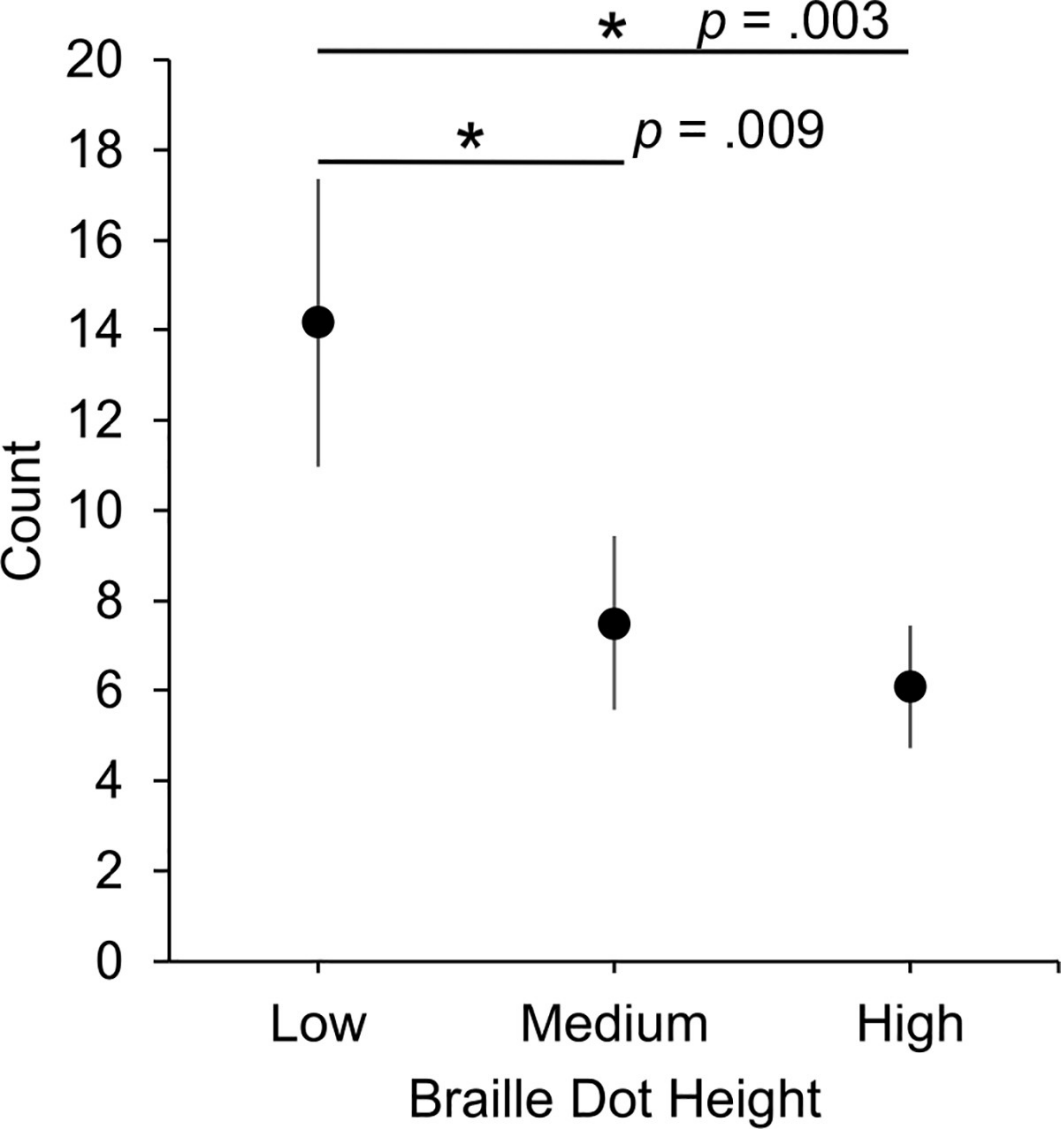
Average number of repeat regressions (with SEMs) at three braille dot heights.

The number of repeat regressions (Fig 4) was significantly affected by braille dot height, χ^2^(2) = 16.76, *p* < .001. Post-hoc pairwise estimated marginal means comparisons indicated that the number of repeat regressions was significantly different between low and medium braille dot heights (*p* = .009, 95% CI [1.20, 12.13]) and between low and high braille dot heights (*p* = .003, 95% CI [2.62, 13.55]), but not significantly different between medium and high braille dot heights (*p* = .509, 95% CI [-4.05, 6.88]).

#### Number of times each word was read

**Fig 5:**
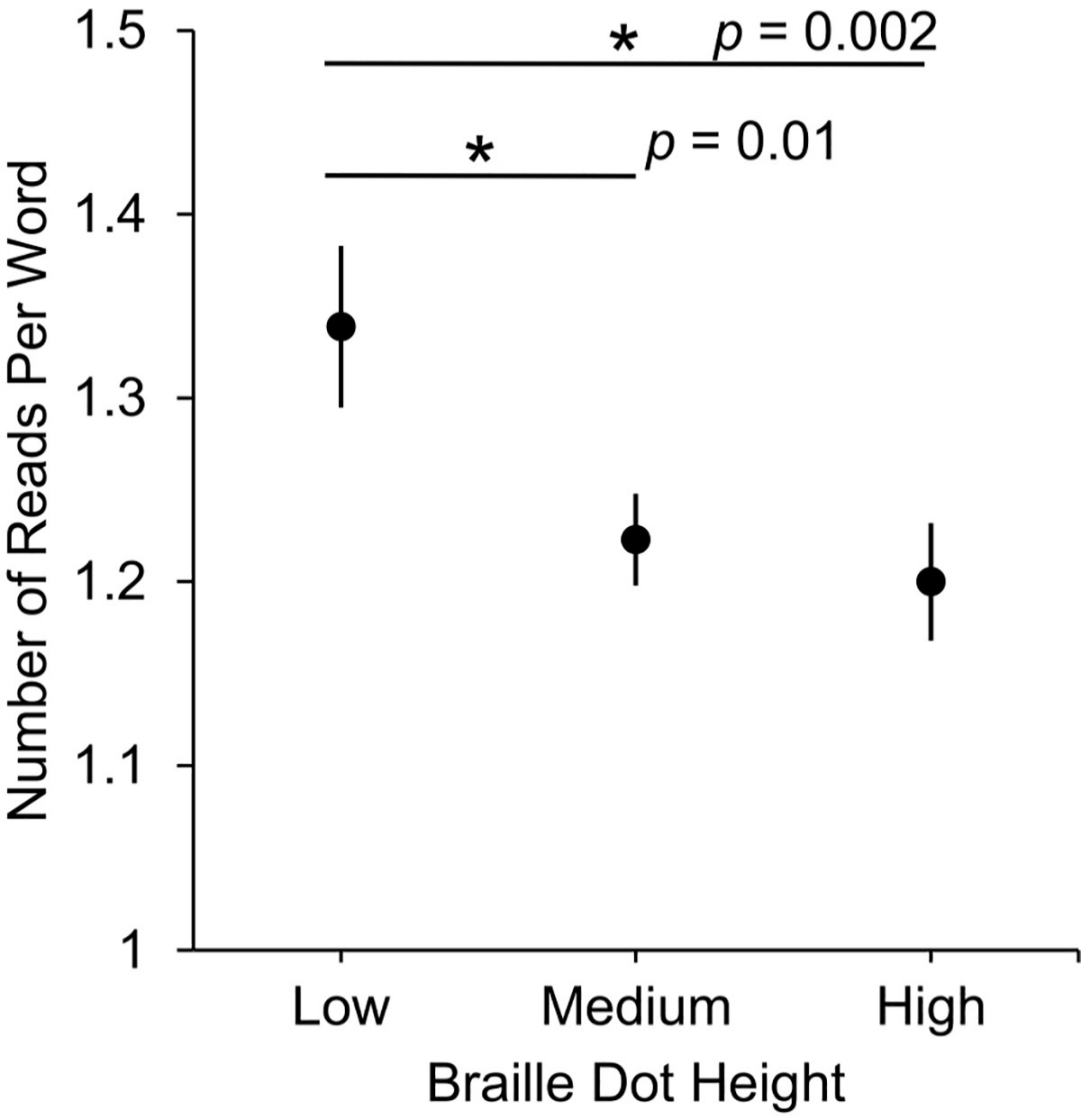
Average number of times each word was read with a distinct forward movement (with SEMs) at three braille dot heights.

The average number of times each word was read (Fig 5) with a distinct forward movement was significantly associated with braille dot height χ^2^(2) = 18.089, *p* < .001. Post-hoc pairwise estimated marginal means comparisons revealed significant differences between low and medium braille (*p* = .010, 95% CI [0.25, 0.21]) and low and high braille (*p* = .002, 95% CI [0.05, 0.23]), but no significant difference between medium and high braille (*p* = .807, 95% CI = [-0.068, 0.11]).

#### Percentage of time rereading

The average percentage of time spent in regressive movements were 11.91% (1.11% SEM), 9.06% (0.73% SEM), and 9.46% (1.43% SEM) for low, medium, and high braille dots respectively. Although there was an increased percentage of time for low braille dot heights compared to the medium and low braille dot heights, the average percent of time spent in regressive movements were not statistically associated with braille dot height χ^2^(2) = 5.05, *p* = .080.

### Movement speeds

**Fig 6:**
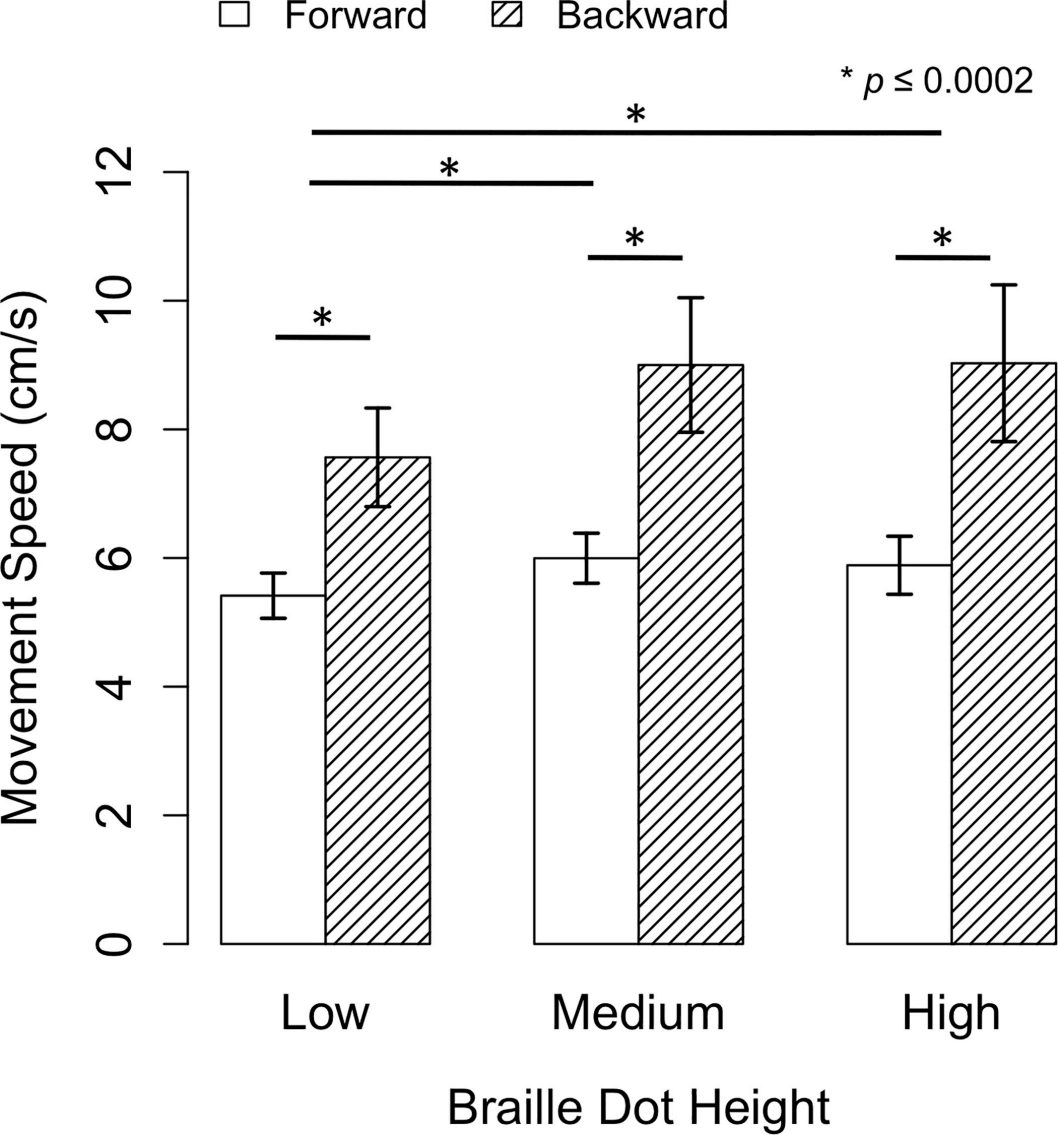
Average forward and backward finger movement speeds (cm/s) with SEMs at three braille dot heights.

The average finger movement speeds (Fig 6) were significantly related to movement direction χ^2^(1) = 172.21, *p <* .001; and were significantly associated with braille dot height χ^2^(2) = 19.52, *p* < .001. However, they were not associated with the interaction of movement direction and braille dot height χ^2^(2) = 0.28, *p* = .871. Post-hoc pairwise comparisons indicated that for each braille dot height condition, backward movement speeds were significantly faster than forward movement speeds (all dot heights: *p* < .0001, 95% CI [0.111, 0.151]). Furthermore, when controlling for movement direction, post-hoc pairwise comparisons indicated that finger movement speeds were significantly different between low and medium braille dot heights (*p* = .0002, 95% CI [0.019, 0.074]) and between low and high braille dot heights (*p* = .0002, 95% CI [0.017, 0.073]), but not between medium and high braille dot heights (*p* = .880, 95% CI [-0.027, 0.024]). On average, backwards movements were 2.77 cm/s faster than forward movements.

## Discussion

In sum, for the low braille dot height condition, we found significantly slower reading rates, significantly more primary regressions, significantly more repeat regressions, and a significant increase in the number of times each word was read compared to medium and high braille dot height. However, there was no significant difference between medium and high braille dot height conditions for those four measures (reading rates, number of primary regressions, number of repeat regressions, and the number of times each word was read).

An interesting question that follows this study is what the tactile threshold value is for humans reading braille. Unfortunately, we could not find a systematic psychophysical determination of the tactile threshold for braille-like dots. For a single dot, it is in the range of only a few microns [51]; however; sensitivity to a single dot is not what is required for reading braille. Instead, we need to consider that lateral interactions with the sensation of adjacent dots are likely to occur while reading braille, especially as the sensing finger is moved.

The tactile height of the dots in the high condition was 0.38 mm, which is near the recommended standard braille dot height and can be assumed to be above the threshold. This assumption is confirmed by the fact that the high dots could be easily read by all of our participants. Our medium dots of 0.18 mm were obviously not near the threshold, since our results were hardly different than the ones obtained in the high dot condition. This height has also been shown to be easily read by many braille readers (71%) [52].

Dots in the low height condition, however, were 0.04 mm, which is likely to be closer to the tactile threshold. This produced an increased frequency of regressive finger movements and diminished reading rate. This finding is confirmed by the fact that braille dots of 0.037 mm height printed with a UV ink-jet layering technique can still be read by blind and visually-impaired individuals [53].

It is important for us to relate our stimuli to the threshold height in the presence of adjacent dots. Note that we need to know the critical tactile height of the dots, not the “tactile acuity” as described by [54], which determines the minimum separable distance between two adjacent dots for them to be perceptually distinct. “Tactile acuity” is not relevant in the context of this study, since braille dots are always an equal distance away from each other in one cell (2.3 mm, a standard set by the Library of Congress [45]). This is well-adjusted to the finding that the maximum response of mechano-receptive afferents to dot arrays is highest between 2 and 3 mm spacing for the two most important tactile receptor types [55].

Lateral interactions also play a role in experiments testing tactile “hyperacuity”, where the lateral displacement of the middle one in a row of three dots must be detected. If no finger movements are allowed, the threshold is very high, at a mean of 0.31 mm (± 0.05 SEM) [56]. If the finger pad is moved over a stationary dot, however, the threshold drops considerably [57]. This condition is relevant here, because we can assume that there will always be finger pad movements while reading braille as in our experiments.

In summary, we conclude that our low braille dot height was close enough to the tactile threshold to have an effect on reading rate and frequency of regressions, but not below the threshold, since our participants could still accurately read the braille text. Our results showed that regressive finger movements during braille reading can be elicited by low levels of stimulus intensity, the same way that has been shown for visual input and reading print.

It is a well-documented fact that regressions occur in the realm of reading print as well as reading braille. Due to the incompatibilities in the physical and physiological mechanisms, the steps involved require a detailed examination to pinpoint commonalities and differences.

Reading is a dynamic process that depends on the intention to acquire information by comprehending text. This requires movement from beginning to end of the text in both media. In the case of reading print, the eye progresses through the text with saccadic eye movements, while the information is acquired during the holding positions between the fast, step-like movements. In the case of reading braille, the information is continuously acquired while the reading finger slides relatively slowly and continuously over raised dots that are arranged in braille cells.

Reading braille with one’s fingertips is an involved task that requires movement of one’s entire hand across the page. Braille readers are aware of their regressive movements, since they can be admonished to avoid them [58]. However, that might not be the case for the fast regressive eye movements during reading print. Given the decision to continue reading, the process is perpetuated until the goal is reached. Nevertheless, a fundamental question that must be addressed repeatedly in both media, which is determining which *direction* the next movement is to go. If the reader is sure enough that the information is sufficiently clear to allow an unequivocal interpretation of the content, the movement will progress in the reading direction. Conversely, if the reader is unsure about the content, the next movement can go in the other direction to re-examine input that has been read before.

The functional role of this point in time has been recognized as fundamental and has received much attention in the published models of the reading process. This strategic point has been described as a moment of “uncertainty” [8, 40, 14, 59–60] or as “ambiguity” [61, 34, 8–9, 62, 59]. This point in time can be seen as an implementation of an informational threshold that determines whether the ultimate tool of resolving it should be used to reduce uncertainty (or entropy) to zero [8].

The literature shows that regressive eye movements that occur when reading print can be influenced by linguistic elements of the reading material (word frequency, word length, language complexity; see [63, 64], visuomotor processes [15], as well as the physical quality of the input signal in reading print [8, 37–38]). What the current study adds to the existing knowledge is the fact that the physical quality of the haptic input in reading braille also influences the occurrence of regressions. In summary, this means that the special state of “uncertainty” can be evoked by diminished input quality alone and, thus, trigger the generation of regressive movements during reading in the visual and tactile realms.

As uncertainty obviously plays an important role in reading, it is tempting to speculate whether there is a region in the human brain that could support this kind of decision-making. The critical feature would have to be that it is involved in reading and independent of the input stimulus modality. Such an area has been described as the VWFA (visual word form area), based on imaging experiments on the human brain [65, 66]. This area in the ventral visual pathway is multimodal, receives input from the visual and tactile systems, and is activated when reading in either input modality. Experiments using transcranial magnetic stimulation (TMS) have shown that disruption of VWFA activity can decrease tactile reading accuracy in sighted braille readers [67] and lesions in that area cause pure alexia [68].

It is important to note that three measures of tactile sensitivity (tactile acuity [gap width], line length, and orientation) show a steady decline with age [50]. This opens the possibility that braille reading in our participants might be affected accordingly, especially given their considerable age range (22 to 70 years). The fact that we did not see an age effect in our cohort can be explained by our use of standardized braille cells that are configured at a size that is far above the thresholds measured in the study by Stevens et al. [50]. A further point to consider is the fact that blind braille readers, like all of our participants, have been found to show higher tactile sensitivity than sighted control subjects [57]. Since we did not conduct any comparisons with sighted subjects, this is a concern we can disregard.

To conclude, the results of our experiment demonstrate that the physical characteristics of tactile input can influence behavioral aspects of reading braille. Since this parallels previous findings on reading print, it is surprising that the role of input strength in the generation of reading regressions has not found a place in unified theories of reading [69, 70, 71]. This is relevant, because a role of regressions during reading was hypothesized early on by Bayle [25], who concluded from an eye movement analysis that they “serve a definite purpose in reading”. A recent series of advanced experiments has demonstrated that regressive movements made while reading print are not only normal and acceptable, but actually augment text comprehension [27, 72]. The present study has increased the likelihood that regressive hand movements while reading braille are likely to serve the same function.

Future studies should investigate the influence of systematic decreases of dot height near the braille reading threshold to further assess regressive movements and their influence on reading comprehension. Taken together with an equivalent study of reading print, this could help to further elucidate those aspects of the reading process that are independent of input modality.

## Supporting information

S1 Data Participants’ demographics (sheet titled “demographics”) and participants’ reading rates, regression counts, reading times, and movement speeds at the three dot heights (sheet titled “data”). (XLSX)
